# The Potential Role of Everlasting Flower (*Helichrysum stoechas* Moench) as an Antihypertensive Agent: Vasorelaxant Effects in the Rat Aorta

**DOI:** 10.3390/antiox11061092

**Published:** 2022-05-31

**Authors:** Marta Sofía Valero, Sonia Nuñez, Francisco Les, Marta Castro, Carlota Gómez-Rincón, María Pilar Arruebo, Miguel Ángel Plaza, Ralf Köhler, Víctor López

**Affiliations:** 1 Departamento de Farmacología, Fisiología y Medicina Legal y Forense, Universidad de Zaragoza, 50009 Zaragoza, Spain; macastro@unizar.es (M.C.); parruebo@unizar.es (M.P.A.); aplaza@unizar.es (M.Á.P.); 2 Instituto Agroalimentario de Aragón, IA2, Universidad de Zaragoza-CITA, 50830 Zaragoza, Spain; fles@usj.es (F.L.); cgomez@usj.es (C.G.-R.); 3 Facultad de Ciencias de la Salud, Universidad San Jorge, Villanueva de Gállego, 50830 Zaragoza, Spain; snunez@usj.es; 4 Instituto de Investigación Sanitaria Aragón (IIS Aragón), 50009 Zaragoza, Spain; 5 Instituto Aragonés de Ciencias de la Salud (IACS), Agencia Aragonesa de Investigación y Desarrollo (ARAID), 50009 Zaragoza, Spain; kohler@araid.es

**Keywords:** antioxidants, calcium channels, hypertension, medicinal plants, NOS, polyphenols

## Abstract

*Helichrysum stoechas* (L.) Moench (*H. stoechas*) is a medicinal plant traditionally used in the Iberian Peninsula to treat different disorders such as arterial hypertension. The aim of this study was to investigate the vascular effects of a polyphenolic methanolic extract of *H. stoechas*, which has high antioxidant activity, and its mechanism of action. Isometric myography studies were performed in an organ bath with rat aortic rings with intact endothelium. The *H. stoechas* extract produced vasorelaxation in the aortic rings that were precontracted by phenylephrine or KCl. L-NAME and Rp-8-Br-PET-cGMPS but not indomethacin or H-89; it also reduced the relaxant response evoked by *H. stoechas* extract on the phenylephrine-induced contractions. *H. stoechas* extract reduced the response to CaCl_2_ similar to verapamil and reduced the phenylephrine-induced contractions comparable with heparin. TRAM-34, apamin and glibenclamide reduced relaxation induced by the *H. stoechas* extract. The combination of L-NAME+TRAM-34+apamin almost completely inhibited the *H.* *stoechas*-induced effect. In conclusion, the relaxant effect of the *H. stoechas* extract is partially mediated by endothelium through the activation of the NO/PKG/cGMP pathway and the opening of Ca^2+^-activated K^+^ channels. Furthermore, the decrease in the cytosolic Ca^2+^ by the inhibition of Ca^2+^ influx through the L-type Ca^2+^ channels and by the reduction of Ca^2+^ release from the sarcoplasmic reticulum via the IP_3_ pathway is also involved.

## 1. Introduction

Since ancient times, natural products have been a valuable resource of therapeutic agents for the prevention and treatment of diseases, with many of the current and developing drugs being natural products or derivatives. These natural products have been gaining more and more popularity, being widely used by the population because of their therapeutic effects and because they are affordable [1].

Natural products offer broad chemical and structural diversity, providing a wide range of nutrients and bioactive compounds including vitamins, minerals, fibre and phytochemical components, among others. Phytochemicals are very important from a health-promoting point of view because they are responsible for the medicinal properties of plants and act as functional components; these compounds are natural molecules derived from plants that are nonnutritive but biologically active and associated with the reduction of risk of important chronic diseases. So far, more than 5000 phytochemicals have been identified, but a high percentage remain to be isolated and identified. These compounds are obtained during the secondary metabolism of plants and are generally divided into polyphenols, terpenoids and alkaloids [2,3]. Polyphenols are the phytochemicals that have a broader spectrum of bioactivity, even being considered therapeutic agents with useful applications as medicines, food supplements, cosmetic products, or agrochemicals for food or plant protection.

Numerous studies support that phytochemicals have a beneficial effect on health due to their antioxidant, anti-inflammatory, antimicrobial, antiallergic, antispasmodic, anticancer, cardioprotective, neuroprotective, antidiabetic and antiobesity activity and because they maintain the homeostasis of the intestinal microbiota [3,4,5]. Due to these biological effects, phytochemicals are powerful therapeutic tools; that is why the bioactive compounds of plants must be identified, their biochemical and physiological effects known, as well as their mechanisms of action, and their efficacy and therapeutic safety guaranteed [6].

The genus *Helichrysum* belongs to the Compositae (or Asteraceae) family and is characterized by a large number of species, approximately 500–600, that are widely distributed throughout southern Europe and Africa. Species of this genus have yellow flowers and a rich amount of secondary metabolites, which provide a great variety of bioactive properties. Within this genus, *Helichrysum stoechas* (L.) Moench *(H. stoechas)* is one of the least known. In the Iberian Peninsula, this species, commonly known as everlasting flower (*siempreviva*, *perpetua* or *sol de oro* in Spanish) is used in traditional medicine. Herbal teas of these flowers are used to treat disorders of the cardiovascular, digestive (bloating, hepatic and pancreatic problems) and respiratory systems (influenza and cold) and have anti-urolithiatic and diuretic effects as well [7,8,9,10].

Previous studies have reported antioxidant and antimicrobial properties [11,12] and anti-inflammatory capacity [13] as well as antidiabetic and neuroprotective properties [12]. Recently, this plant has been used to assess the degree of soil contamination in a region of Spain, suggesting its utility for the phytostabilization of contaminated soils [14].

A recent study carried out by our group reported the phytochemical analysis of a metanolic extract of *H. stoechas* and showed a high content of polyphenolic compounds (166.6 μg gallic acid equivalents (GAE)/mg by the Folin-Ciocalteu method) with a particular phenolic profile that was isolated by chromatography and identified using NMR and MS [12]. The main components isolated were arzanol, homodimeric α-pyrone (helipyrone and p-hydroxybenzoic acids), phenolic acids (caffeic acids, neochlorogenic acids and 5-7-dihydroxy-3,6,8-trimethoxyflavone), flavonoids (isoquercitrin, quercetagetin-7-O-glucopyranoside) and diacylglycerol (santinol B). Furthermore, this phenolic profile demonstrated high antioxidant properties [12].

Arzanol pyranone is one of the most characteristic phenols found in this genus, in the extract of both *H. stoechas* and another species known as *H. itallicum* [12]. This bioactive compound explained the different properties such as antioxidant, antiviral (anti-HIV-1), and anti-inflammatory (by inhibiting NF-κB and mPGES-1 activity, as well as the production of pro-inflammatory cytokines) [15,16,17].

It is known that phenolic compounds exert antioxidant activity [18,19,20]. They improve nitric oxide (NO) bioavailability and reduce its degradation [21], so they could have an antihypertensive effect by reducing the production of reactive oxygen species (ROS) and thereby preventing vascular alteration. Studies have shown that polyphenols have a cardiovascular protective effect, improving the functions of endothelial cells due to its antioxidant, anti-inflammatory, antihypertensive, antithrombotic and antiproliferative properties [22,23]. Endothelial dysfunction represents the loss of the endothelium’s ability to modulate vascular tone by releasing relaxation factors. This dysfunction is thought to promote cardiovascular pathologies such as hypertension, atherosclerosis, stroke, heart disease, peripheral vascular disease, diabetes, chronic kidney failure, thrombosis and metastasis [24,25].

In keeping with the potentially beneficial compounds in *H. stoechas* extracts and the lack of mechanistic studies investigating its physiological effects at the vascular level, the objective of this study was to evaluate the effect of a methanolic *H. stoechas* extract on the vascular smooth musculature and its mechanism of action, as well as to study whether these effects are due to arzanol, the main biologically active component of the extract.

## 2. Materials and Methods

### 2.1. Reagents and Chemicals

The preparation of methanolic *H. stoechas* extract was previously described [12], and a plant voucher was deposited at the Universidad San Jorge herbarium (ref. 002–2014). The phenolic profile of this extract was previously determined [12] (see Introduction).

The compositions of the buffers used were as follows: Krebs buffer (in millimolar): NaCl 120, KCl 4.7, CaCl_2_ 2.4, MgSO_4_ 1.2, NaHCO_3_ 24.5, KH_2_PO_4_ 1, and glucose 5.6. Ca^2+^-free Krebs: NaCl 120, KCl 4.7, CaCl_2_ 2.40, MgSO_4_ 1.2, NaHCO_3_ 24.5, KH_2_PO_4_ 1, glucose 5.6, and ethyleneglycoltetraacetic acid (EGTA) 1. Ca^2+^-free high K^+^ Krebs ([K^+^]_o_ = 50 mM). All buffers were adjusted to pH 7.4. Apamin (AP), arzanol, barium chloride dihydrate (BaCl_2_), glibenclamide (Glib), heparin (H), H-89 dihydrochloride hydrate, indomethacine, Nω-Nitro-L-arginine methyl ester hydrochloride (L-NAME), phenylephrine (PE), ruthenium red (RR) and verapamil (V) were obtained from Sigma (Madrid, Spain).

Tetraethylammonium (TEA), 1-[(2-chlorophenyl) diphenylmethyl]-1H-pyrazole (TRAM-34) and Rp-8-Br-PET-cGMPS were purchased from Tocris (Madrid, Spain). AP was diluted in acetic acid. Glib, TRAM-34 and arzanol were dissolved in dimethyl sulfoxide (DMSO). All other chemicals were dissolved in distilled water.

### 2.2. Animals

The care and use of animals were in accordance with the Spanish Policy for Animal Protection RD 53/2013, RD1386/2018 and RD118/2021, which meet the European Union Directive 2010/63 on the protection of animals used for experimental and other scientific purposes. All protocols were approved by the Ethics Committee for Animal Experiments of the University of Zaragoza (Spain) (reference: PI66/17, 18 January 2018). Male Wistar albino rats (200–250 g) were purchased from Janvier (LeGenest St. Isle, France) and were maintained with a standard feed and free access to water. The animals were euthanized by pentobarbital sodium (60 mg/Kg i.p.) followed by cervical dislocation.

### 2.3. Preparation of the Aortic Rings and Isometric Myography

Myography on the rat aorta artery rings was performed as described previously [26]. After sacrifice, the thoracic aorta was removed carefully, placed in ice-cold Krebs buffer, and cleaned of fat and adherent connective tissue. The aorta was cut into rings (3 mm long), and the rings were individually mounted to an isometric force transducer (Pioden UF1, Graham Bell House, Canterbury, UK) for tension measurement. The organ bath contained 5 mL Krebs buffer maintained at 37 °C and gassed with 95% O_2_–5% CO_2_. Tension was recorded and digitalized at a sample rate of 0.5 samples per second using a data acquisition system (ED410 e-corder 410, eDAQ, Cibertec, Madrid, Spain). An initial tension of 1 g was applied to the preparations to achieve spontaneous tone, and rings were allowed to equilibrate for 60 min with changing the bath buffer every 20 min.

### 2.4. The Experimental Protocols

After the stabilization of spontaneous tone, PE (1 µM) or KCl (80 mM) were used to induce sustained contractions. Fifteen minutes thereafter, the effects of *H. stoechas* extract, methanol (MeOH, solvent of the extract), arzanol and DMSO (solvent of the arzanol) were assessed, and cumulative concentration-response curves were constructed on endothelium-intact aortic rings. The vasorelaxant effect was calculated as the percentage change of precontraction to basal tone in the absence of the contractile agent.

To specifically measure endothelium-mediated vasorelaxation, the NO-synthase blocker L-NAME (10 µM) and the cyclooxygenase blocker indomethacin (10 µM) were added to the bath 20 min prior to the addition of PE. At stable PE-induced contraction, a concentration-response curve for the *H. Stoechas* extract was performed.

Next, the involvement was investigated of secondary messengers such as cAMP and cGMP and potassium channels in response to the *H. stoechas* extract. 20 min before addition of PE (1 µM), aortic rings were preincubated with H-89 (200 nM), Rp-8-Br-PET-cGMPS (10 µM), TRAM-34 (1 µM), AP (1 µM), Glib (10 µM), BaCl_2_ (30 mM) and TEA (tetraethylammonium, 0.5 mm). At stable contraction, the *H. stoechas* extract (0.3 and 1 mg/mL) was added for 15 min. The relaxant effect was compared with the response obtained by control (*H. stoechas* extract alone).

To examine the effects of *H. stoechas* extract on PE-induced contraction, the rings were preincubated with the extract or the solvent for 20 min, and a PE concentration-response curve was made. The contractile response of each concentration was compared to its control (baseline).

The role of Ca^2+^ influx in relaxation to the *H. stoechas* extract was studied. After prior incubation with Krebs buffer, the medium was replaced with Ca^2+^-free Krebs buffer for 15 min and then with Ca^2+^-free high-K^+^ buffer. Then, the rings were preincubated with methanol, *H. stoechas* extract (0.3 and 1 mg/mL) or V (1 µM) for 15 min, and after that time, cumulative concentration-response curves for CaCl_2_ (10^−5^–10^−2^ M) were constructed. The responses obtained to CaCl_2_ in the presence of methanol served as control.

In order to know if the response of the extract involves intracellular Ca^2+^, we evaluated the effect of the *H. stoechas* extract on the contractile response of PE (1 µM). For this, the rings were incubated for 15 min with verapamil (V) and the combination of V with RR and H before the addition of PE. Contractions were compared with controls.

Each protocol was performed on aortic rings from six or eight different animals.

### 2.5. Analysis of the Data

Data are expressed as mean ± standard error of mean (SEM). Statistical significance was tested using one-way analysis of variance (ANOVA) followed by the Dunnett test (when parametric distribution is observed), Kruskal-Wallis test (nonparametric) and paired two-tailed Student’s t test, if appropriate. A two-way ANOVA was performed to assess the interaction between two variables and the effects of each one, separately. *p* values <0.05 were considered statistically significant. The concentration of compound that inhibited 50% of the maximal contraction (EC_50_) was calculated as the geometric mean with 95% confidence intervals (CI). All statistical analyses were performed and all figures were created with GraphPad Prism 6.

## 3. Results

### 3.1. The Effects of H. stoechas Extract on PE- and KCl-Induced Contractions

**Figure 1:**
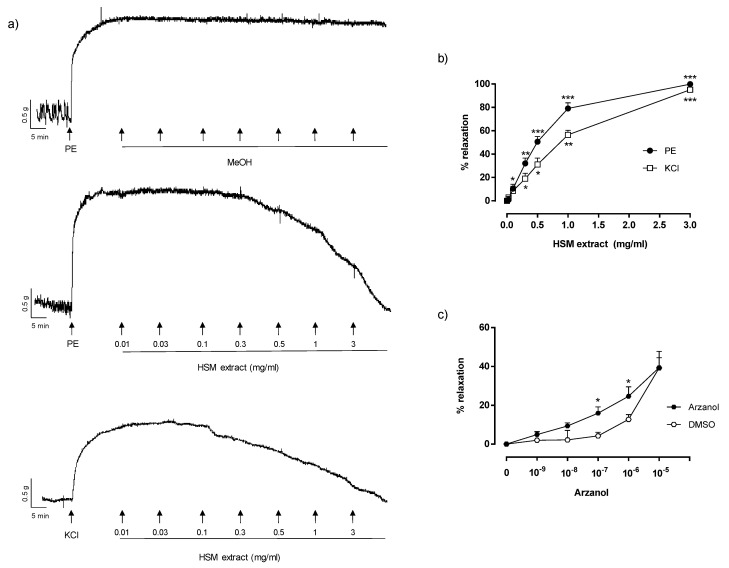
(**a**) Representative recordings showing the precontractions evoked in intact isolated rat aorta rings by PE (1 µM) or KCl (80 mM) and the effects induced by MeOH and *H. stoechas* extract (HSM; 0.01–3 mg/mL) on these contractions. (**b**) The relaxant effects of different concentrations of extract (mg/mL) on PE- or K+-induced contractions of isolated rat aorta rings (PE, F = 219, *p* < 0.0001; KCl, F = 187.6, *p* = 0.0001). * *p* < 0.05, ** *p* < 0.01 and *** *p* < 0.001 vs. control values (basal tone). (**c**) The relaxant effects of arzanol and DMSO on PE-induced contractions of isolated rat aorta rings. * *p* < 0.05 vs. DMSO values. Data points are mean ± SEM (*n* = 6–8).

PE (1 µM) or KCl (80 mM) induced a sustained contraction over time in the rat aortic rings. The *H. stoechas* extract induced a concentration-dependent vasorelaxation in the PE- or KCl-precontracted rings, with EC_50_ of 0.49 mg/mL (0.47–0.55, 95% CI) and 0.87 mg/mL (0.77–0.99, 95% CI), respectively. However, at the maximum concentration, 3 mg/mL, the extract produced a similar relaxation in the PE- or KCl-precontracted rings (PE: 99.9 ± 1.9% vs. KCl: 95.1 ± 1.4%) (Figure 1a,b). To exclude the possibility that the effect of the extract was caused by the solvent (MeOH), we studied the per se effect of MeOH in the precontracted rings and found that at the concentrations used, MeOH did not produce changes in muscle tone (Figure 1a). 

To test whether the relaxing effect of *H. stoechas* extract was produced by one of its most characteristic components, an arzanol-concentration-response curve was performed (10^−9^ M–10^−5^ M) (Figure 1c). To exclude the possibility that the relaxing effect was caused by the per se relaxing effects of the solvent DMSO, we tested the same concentrations of DMSO (Figure 1c). The greatest relaxing effect of arzanol was found at a concentration of 10^−5^ M. Arzanol showed a stronger vasorelaxant effect than DMSO at concentrations of 10^−7^ and 10^−6^ M. However, at the highest concentration tested (10^−5^ M), arzanol showed the same effect as that obtained by the corresponding concentration of the solvent alone. Considering these results, arzanol was discarded as the main phenolic involved in the vasorelaxant effects, with other compounds being responsible for the observed activity.

### 3.2. The Effects of Indomethacin and L-NAME on H. stoechas Extract-Induced Vasorelaxation

**Figure 2:**
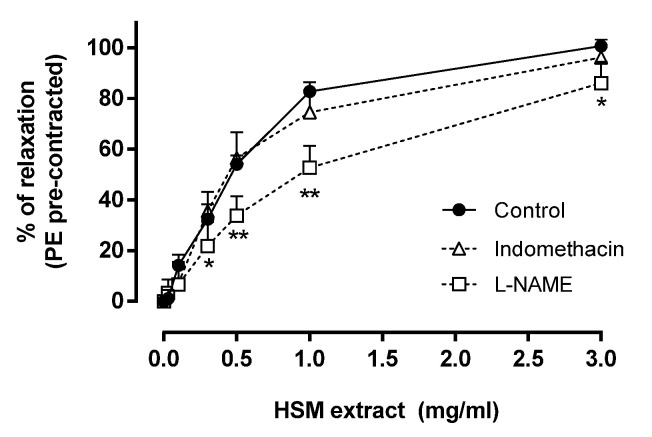
The relaxant effects of increasing concentrations of *H. stoechas* (HSM) extract in the absence (control) and presence of L-NAME (10 µM) or indomethacin (10 µM) on PE-induced contractions evoked in isolated rat aorta rings. Data points are mean ± SEM (*n* = 6–8). * *p* < 0.05 and ** *p* < 0.01 vs. control.

To study the involvement of endothelium-derived factors in the relaxation evoked by the *H. stoechas* extract on the PE-contracted rings, intact aortic rings were preincubated with the nitric oxide synthase inhibitor L-NAME (10 µM) and with the nonselective cyclooxygenase inhibitor indomethacin (10 µM). As shown in Figure 2, indomethacin had no effect on the *H. stoechas* extract-induced vasorelaxation on rings contracted with PE. However, preincubation with L-NAME significantly inhibited *H. stoechas* extract-induced vasorelaxation compared with control. The maximum blocking response to the relaxing effect of *H. stoechas* extract by L-NAME was 30% at the concentration of 1 mg/mL (Figure 2).

### 3.3. The Effects of PKA and PKG Inhibitors on H. stoechas Extract-Induced Vasorelaxation

**Figure 3:**
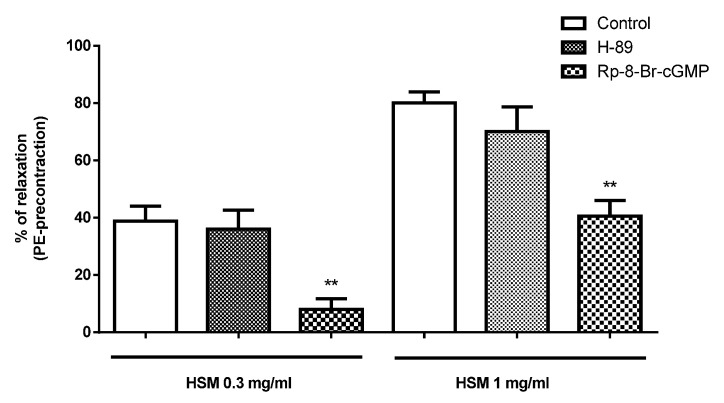
The relaxant effects of *H. stoechas* extract (HSM; 0.3 and 1 mg/mL) in the absence (control) and presence of the protein kinase A inhibitor H-89 (200 nM) or the protein kinase G inhibitor Rp-8-Br-cGMP (10 µM) on PE-induced contractions evoked in isolated rat aorta rings (Interaction: F = 0.32, *p* = 0.7277; Dose: F = 52.40, *p* < 0.0001, Way: F = 18.42, *p* < 0.0001). Data points are mean ± SEM (*n* = 6–8). ** *p* < 0.01 vs. control.

Figure 3 shows the vasorelaxant responses to different concentrations of *H. sotechas* extract (0.3 and 1 mg/mL) in aortic rings precontracted with PE (control) and PE-precontracted rings in the presence of the protein kinase A inhibitor H-89 (200 nM) or the protein kinase G inhibitor Rp-8-Br-cGMP (10 µM). While Rp-8-Br-cGMP significantly reduced the vasorelaxation by 79.3% and 50.64% at 0.3 and 1 mg/mL *H. stoechas* extract, respectively, H-89 had no effect on the response.

### 3.4. The Effects of H. stoechas Extract on the Contractile Response to PE

**Figure 4:**
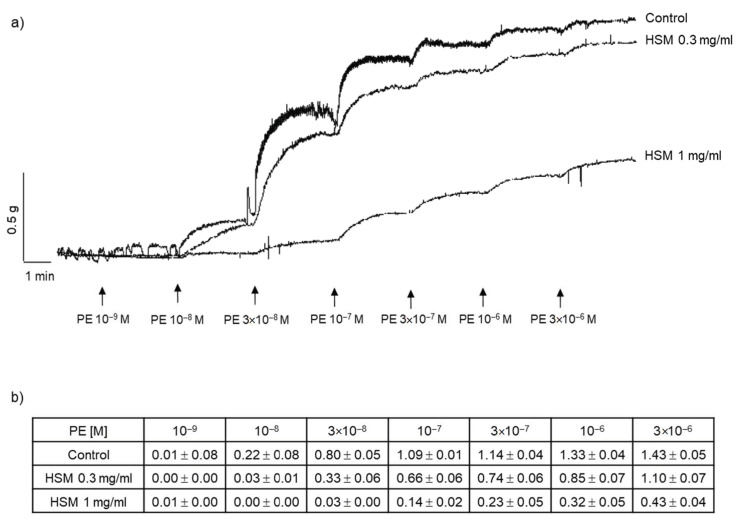
The contractile response to PE after the incubation of the rings with methanol and *H. sotechas* extract (HSM). (**a**) Representative recordings showing the contractions produced by PE (10^−9^–3 × 10^−6^ M) in rings preincubated with methanol (control) or HSM (0.3 and 1 mg/mL). (**b**) The tension (g) produced by each concentration of PE. Data are expressed as mean ± SEM (*n* = 3–6).

To assess the effect of the *H. stoechas* extract on a contractile agonist, we preincubated the rings with the extract (0.3 and 1 mg/mL) and then performed a cumulative PE curve (10^−9^–3 × 10^−6^ M). The contractile response of PE decreased in a concentration-dependent manner (Figure 4). Thus, preincubation with *H. stoechas* extract at 0.3 and 1 mg/mL reduced the contraction induced by the highest concentration of PE by 23% and by 70% respectively, compared with the contraction in absence of the extract (Figure 4).

### 3.5. The Effects of Ca^2+^ Influx on H. stoechas Extract-Induced Vasorelaxation

**Figure 5:**
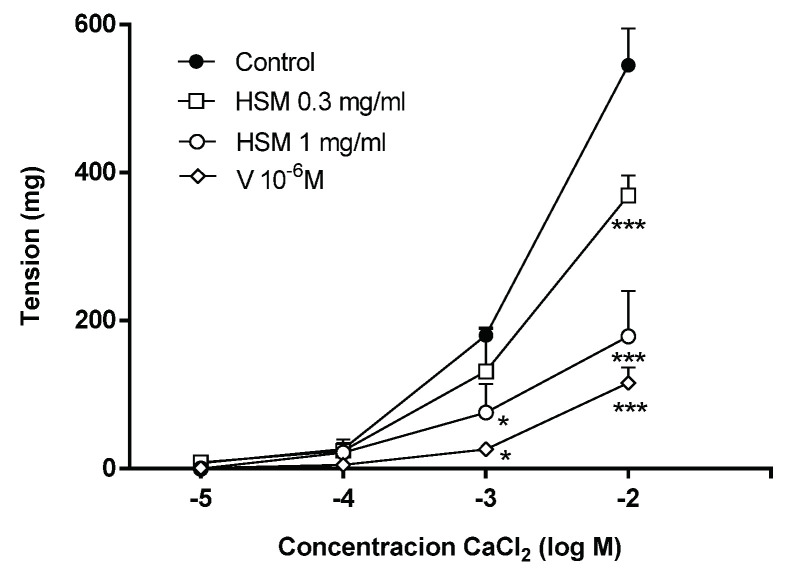
The effects of preincubation with *H. stoechas* extract (HSM, 0.3 or 1 mg/mL) or verapamil (V, 10^−6^ M) on tension produced by different concentrations of CaCl_2_ in rat aorta. Data points are mean ± SEM (*n* = 6–8). * *p* < 0.05 and *** *p* < 0.001 vs. control (HSM extract solvent alone).

To examine the role of extracellular Ca^2+^ in the vasorelaxation induced by *H. stoechas* extract, the aortic rings were preincubated in a Ca^2+^-free K^+^-rich buffer with the *H. stoechas* extract at 0.3 or 1 mg/mL or verapamil, a Ca^2+^ channel blocker. After the incubation period, a cumulative Ca^2+^ curve was performed by adding increasing concentrations of CaCl_2_. As a control, the rings were preincubated with the solvent. The *H. stoechas* extract at 0.3 and 1 mg/mL reduced the maximum response to CaCl_2_ by 32.3% and 67.1%, respectively (Figure 5). Verapamil at 10^−6^ M produced a similar relaxation of 78.7% (Figure 5).

### 3.6. Role of Intracellular Ca^2+^ on the Effect of H. stoechas Extract on the Contractile Response Induced by Phenylephrine

**Figure 6:**
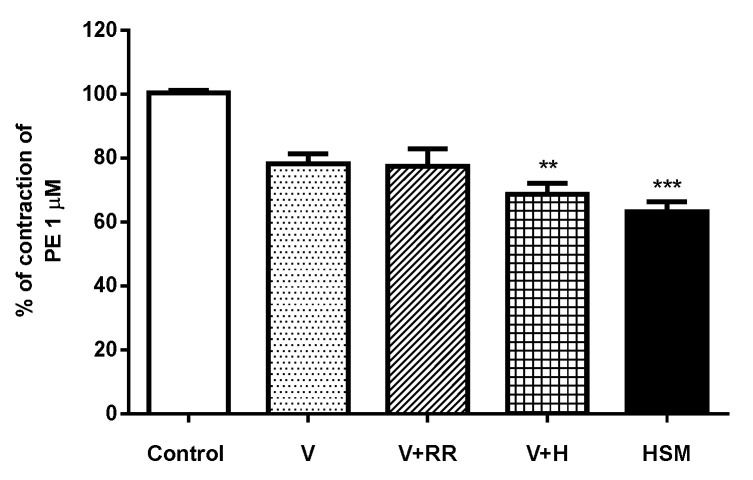
The effects of preincubation with verapamil (V, 1 µM), the combinations of V with ruthenium red (10 µM) (V + RR) or V with heparin (50 mg/mL) (V + H), and *H. stoechas* extract (HSM, 3 mg/mL) on contraction to PE (1 µM) in rat aorta. The Kruskal-Wallis test was performed. Data points are mean ± SEM (*n* = 6–8). ** *p* < 0.01 and *** *p* < 0.001 vs. control (response alone to PE).

In a series of experiments, we evaluated whether intracellular Ca^2+^ release is involved in the vasorelaxant response to *H. stoechas* extract. For this, aortic rings were preincubated with the L-type Ca^2+^ channel blocker verapamil and the ryanodine receptor inhibitor, ruthenium red or verapamil and the inositol 1,4,5-triphosphate (IP_3_) receptor inhibitor heparin before the addition of PE by ≈22% (control). This response was not modified when ruthenium red was added along with verapamil. However, the addition of heparin induced a greater reduction in the contractile response to PE (31.7%). The decrease in the contractile response to PE in the presence of verapamil and heparin was similar to that produced by 3 mg/mL of *H. stoechas* extract (36.9%) (Figure 6).

### 3.7. The Effects of K+ Channels Inhibitors on the H. stoechas Extract-Induced Vasorelaxation

The role of K^+^ channels in the relaxing response to *H. stoechas* extract was studied by preincubating the rings with various K^+^ channel blocking agents such as TRAM-34 (1 µM) or apamin (AP, 1 µM) or selective inhibitors of Ca^2+^-activated K^+^ channels of either intermediate (IK_Ca_) or small-conductance (SK_Ca_): respectively, glibenclamide (Glib, 10 µM), an inhibitor of ATP-sensitive K^+^ channel (K_ATP_); TEA (0.5 mM), a nonspecific K^+^ channel inhibitor; and barium chloride (BaCl_2_, 30 mM), a nonspecific inhibitor of inward rectifier K^+^ channels (K_IR_).

**Figure 7:**
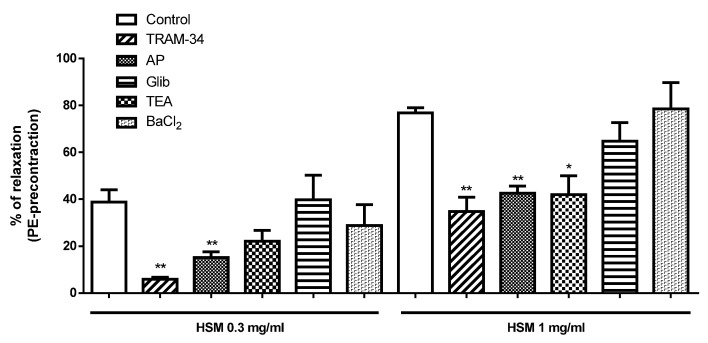
The effects of TRAM-34 (1 µM), apamin (AP, 1 µM), glibenclamide (Glib, 10 µM), tetraethylamonium (TEA, 0.5 mM) and BaCl_2_ (30 mM) on the vasorelaxant response of *H. stoechas* extract (HSM, 0.3 and 1 mg/mL) in isolated rat rings precontracted with PE (Interaction: F = 0.68, *p* = 0.6426; Dose: F = 11.08, *p* < 0.0001, Way: F = 61.59, *p* < 0.0001). Data points are mean ± SEM (*n* = 6–8). * *p* < 0.05 and ** *p* < 0.01 vs. control.

The vasorelaxant effects of the *H. stoechas* extract at 0.3 and 1 mg/mL were inhibited by TRAM-34 (84.7% and 54.8%, respectively) and AP (61% and 44.6%, respectively). Glib significantly reduced the vasorelaxant effect only at the highest concentration of extract (45.3%). In contrast, TEA and BaCl_2_ did not affected the vasorelaxation response of the *H. stoechas* extract (Figure 7). At the concentrations used, the K^+^ channels inhibitors did not show any per se effect on the basal muscle tone.

### 3.8. The Inhibition of H. stoechas Extract-Induced Vasorelaxation by the Combination of TRAM-34 and Apamin with or without L-NAME

We investigated possible additive effects of the combination of the K^+^ channel inhibitors (TRAM-34 and AP) and the NO inhibitor, L-NAME, on the relaxant response evoked by the *H. stoechas* extract.

**Figure 8:**
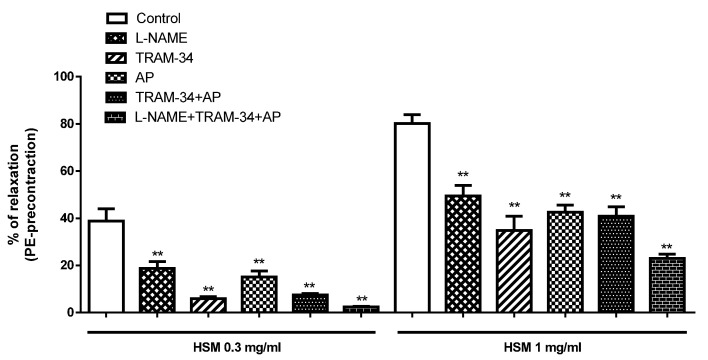
The effects of preincubation with L-NAME (10 µM), TRAM-34 (1 µM), apamin (AP, 1 µM) and the combination of TRAM-34+AP and L-NAME+TRAM-34+AP on the vasorelaxant response evoked by *H. stoechas* (HSM; 0.3 and 1 mg/mL) in isolated rat rings precontracted with PE (HSM 0.3 mg/mL; F = 51.45, *p* < 0.0001 and HSM 1 mg/mL; F = 48.19, *p* < 0.0001). Data are mean ± SEM (*n* = 6–8). ** *p* < 0.01 vs. control.

The most interesting result was that the combination of TRAM-34 and AP did not have additive effects on the vasorelaxation produced by the extract. However, L-NAME showed additive effects in the presence of these K^+^ channel inhibitors (Figure 8).

## 4. Discussion

*H. stoechas* (L.) Moench is used in traditional medicine for the treatment of various pathologies, and especially those of the cardiovascular system. Until now, in vitro studies have shown that this plant has strong antioxidant and anti-inflammatory activities and antimicrobial capacity as well as antiproliferative and antidiabetic effects [12]. Recently, neuroprotective effects were observed in in vitro and in vivo studies [12,27]. However, the vascular activity of *H. stoechas* is unknown.

Here, we demonstrated for the first time the capacity of a methanolic extract of *H. stoechas* to relax rat aortic rings, revealing its vascular mechanisms of action. According to our data, the vasorelaxation depended on the activation of the NO/PKG/cGMP pathway, opening of K^+^ channels and inhibition of Ca^2+^ signalling.

Our results show that the *H. stoechas* extract counteracts contraction to PE (an α1-adrenergic agonist) and KCl (a classical membrane depolarizing agent) in endothelium-intact aortic rings. The relaxant effect of the *H. stoechas* extract on PE-induced contraction was reduced by the inhibition of NO synthesis but not by inhibition of cyclooxygenase activity. In addition, preincubation with the cGMP inhibitor Rp-8-Br-cGMP blocked the vasorelaxant effect of the extract, supporting that the NO and cGMP pathways are involved mechanistically. In contrast, protein kinase A activity did not seem to be required. These results demonstrated that the vasorelaxant effects of the *H. stoechas* extract on rat aortic rings involve the NO/PKG/cGMP pathway and are thus partly endothelium dependent.

It is well-known that the endothelium plays an important role in maintaining vascular homeostasis through the secretion and release of vasodilator substances such as NO and prostacyclin as well as endothelium-dependent hyperpolarization (EDH) [24,25,28]. EDH is an additional major endothelial vasodilator system that acts in many vessels and species mainly through the opening of endothelial KCa channels, causing hyperpolarization. This potential change spreads through presumably direct electrical gap-junctional coupling, either K^+^-release or the smooth-muscle K^+^ channel activating diffusible factors to the smooth muscle and closes VDCCs, causing a decrease in cytosolic Ca^2+^ and, finally, vasodilation. So, K^+^ channels also play a central role in the regulation of vascular smooth muscle tone [29,30]. In this study, we show that TRAM-34 and AP, selective inhibitors of endothelial Ca^2+^-activated K^+^ channels of intermediate (IK_Ca_) and small conductance (SK_Ca_) respectively, significantly decreased the relaxant response to the *H. stoechas* extract in these rat aortic rings. Glib, an inhibitor of ATP-sensitive K^+^ channels (K_ATP_), only reduced the response at the highest concentration of the extract tested. However, TEA, a nonspecific inhibitor of K^+^ channels, and BaCl_2_, an inhibitor of inward rectifier K^+^ channels (K_IR_) and the large-conductance K^+^ channels expressed in smooth muscles, did not alter the response to the *H. stoechas* extract. These findings implicate the endothelial K^+^ channels IK_Ca_ and SK_Ca_ and, to a lesser extent, K_ATP_ in the relaxing response to the *H. stoechas* extract. Therefore, the EDH mechanism also contributes to the *H. stoechas*-induced vasorelaxation.

It is noteworthy that in addition to initiating the EDH vasodilator response, endothelial SK_Ca_ and IK_Ca_ also contribute to the generation of NO, presumably by promoting endothelial signalling [28,31]. This occurs because these channels produce hyperpolarization of the endothelial cell, which induces an influx of Ca^2+^ through voltage-independent Ca^2+^ channels. This cation binds to NOS through calmodulin, which activates and thereby induces the synthesis of NO by this enzyme [31,32]. Thus, the effect of the combination of the SK_Ca_ and IK_Ca_ inhibitors together with or without the NO release inhibitor was assessed. Treatment with TRAM-34 and apamin in the presence of NOS inhibited the effect induced by the *H. stoechas* extract in a stronger way than in the absence of NOS, showing that hyperpolarization and NO release are additive.

In vascular smooth muscle, Ca^2+^ mobilization has an important role in the regulation of contraction and relaxation. Cytosolic Ca^2+^ results from Ca^2+^ influx from the extracellular space through receptor-operated Ca^2+^ channels (ROCCs) or VDCCs and from the release of Ca^2+^ from intracellular stores [32,33]. In our experiments, *H. stoechas* extract seemed to affect both Ca^2+^ pathways and by this reduced the contractile response to KCl and PE. The contraction produced by KCl in VSMCs is caused by the opening of VDCCs and therefore by the entry of Ca^2+^ from the extracellular medium, whereas the contraction caused by PE is due to both the influx of extracellular Ca^2+^ through ROCCs and the release of intracellular Ca^2+^ from the SR after the activation of IP_3_ receptors [33,34]. At low concentrations, the extract showed a greater vasorelaxant effect in the rings precontracted with PE than in those precontracted with KCl (see Figure 1), suggesting that the *H. stoechas* extract acts as an inhibitor of Ca^2+^ efflux from intracellular stores. In addition, the preincubation of the rings with the extract strongly reduced the contractions produced by increasing concentrations of PE in a concentration-dependent manner.

Furthermore, the *H. stoechas* extract inhibited the contraction caused by the influx of extracellular Ca^2+^ evoked by the addition of increasing concentrations of CaCl_2_ to a Ca^2+^-free medium. This vasorelaxant effect induced by the highest concentration of the *H. stoechas* extract was similar to that produced by verapamil, an inhibitor of L-type voltage-dependent Ca^2+^ channels. This finding suggests that the *H. stoechas* extract inhibits Ca^2+^ influx into smooth muscle cells through the VDCCs and thus reduces Ca^2+^-dependent contraction. On the other hand, our results suggest that *H. stoechas* extract also blocks intracellular Ca^2+^ release from the SR. Thus, the *H. stoechas* extract reduced the PE-evoked contraction in a similar manner to that produced by heparin, an IP_3_ receptor inhibitor, suggesting that the vasorelaxant response to *H. stoechas* extract involves the inhibition of the IP_3_ signalling pathway. All these results suggest that a mechanism of action by which the *H. stoechas* extract produces vascular relaxation is the inhibition of vascular smooth muscle Ca^2+^ signalling by blocking both Ca^2+^ entry and release from SR. Together, the *H. stoechas* extract uses multiple and likely additive endothelial and vascular smooth muscle mechanisms to produce vasorelaxation in this vessel.

To shed additional light on the active components in the extract, we studied whether a main component, arzanol, mimics the effect of the extract. However, we found that arzanol was only responsible for a small percentage of the vasorelaxant effect produced by the *H. stoechas* extract, suggesting that in addition to arzanol, other not yet identified vasorelaxing polyphenols or other components are involved here.

Concerning phenols, most of them have been reported to cause blood vessel dilation predominantly through activation of the NO/cGMP pathway. For instance, a study that analysed the direct relationship between NO release and the relaxant effect on porcine coronary rings induced by plant phenols showed that endothelium-dependent vasorelaxation is limited to those compounds that induce nitric oxide elevation > 5 nM (=Km value of soluble guanylate cyclase). Caffeic acid and isoquercitrin, compounds present in our extract, moderately increased the formation of NO (5 nM < [NO] < 8.5 nM) [35]. In this study, the vasorelaxant effect was significantly attenuated after treatment with L-NAME, and relaxations were almost completely abolished on the endothelium-denuded rings. To the contrary, indomethacin was without effect.

In the same way as our extract, the flavonoid isoquercitrin induces both endothelium-dependent and endothelium-independent vasodilation in the arteries of the mesenteric vascular bed of rats, through the release of NO but not PGI_2_ and through the opening of K^+^ channels in the cells of the smooth muscle [36]. Another study showed that caffeic acid exerted a vasorelaxant effect in isolated rat thoracic aorta precontracted with PE and KCl. This effect was partially dependent on NO and on blocking the increase in cytosolic Ca^2+^ elicited by Ca^2+^ influx and from intracellular stores [37].

Still, the phenol or component(s), responsible for the vasorelaxation capability of *H. stoechas* reported here remain to be elucidated in future studies.

In phytopharmacology, polyphenols are considered beneficial for relieving harmful oxidative stress and enhanced contractility in the vasculature. This enhanced contractility caused by ROS probably relies on increased cytosolic Ca^2+^ in VSMCs as a result of excessive Ca^2+^ influx through Ca^2+^ channels and release from the SR; inhibition of the SERCA pump; and other impairments like modification of cytoskeletal proteins [33]. It can be speculated that antioxidant compounds, like those that are present in our extract [12], could relieve oxidative and hypercontractility by counteracting excessive Ca^2+^ signaling and could therefore exert a cardiovascular protective effect.

Although there are no studies on the cardioprotective and vascular effects of *H. stoechas*, there are studies with other species of the same genus that suggest such cardioprotective utility. For instance, a crude aqueous extract of the root or leaves of *Helicrysum ceres* in rats induced hypotension and natriuresis [38]. Moreover, ethanolic extract of *Helichrysum ceres* leaves lowered blood pressure by producing negative inotropic and chronotropic effects in vivo experiments and evoked vasorelaxant effects in vascular tissue in isolated aortic rings involving both endothelium-dependent and endothelium-independent mechanisms [39]. The oral administration of aqueous and ethanolic extracts of *Helichrysum plicatum* (500 mg/Kg/bw) and ethanolic extracts of *Helichrysum graveolens* (500 mg/Kg/bw) showed significant antihyperglycaemic and antioxidant activity in streptozotocin-induced-diabetic rats, possibly preventing the onset of cardiovascular pathologies [40,41]. The study of flavonoids from the flowers of *Helichrysum arenarium* showed anti-atherosclerotic activities and fewer anti-inflammatory molecules [42]. Additionally, other plants in the Asteraceae family have been extensively studied and suggested to improve cardiovascular health, at least in part because they show vasodilator action involving pathways similar to those used by our extract [43]. In more detail, an aqueous extract of *Artemisia campestris* L. aerial parts showed a vasorelaxant effect on isolated rat aorta by activating the calmodulin-NO-cGC-PKG pathway and evoked a decrease in intracellular calcium by blocking VOCC channels and activating the SERCA pump. Kir, K_ATP_, Kv and nonspecific K+ channels were not involved here [44]. Another study showed that organic extracts of *Achillea millefolium* produced vasodilation through endothelium-dependent NO release by increasing cGMP levels and by calcium channel blockade [45]. An aqueous extract of leaves of *Tridax procumbens* causes vasodiltation in the isolated rat aortic artery though the NO-cGMP and cAMP pathways and the dephosphorylation of MLC [46]. An ethanolic extract of *Vernonia amygdalina* Del. showed a pronounced vasorelaxant effect by activating the NO/cGMP and PGI_2_ pathways; reducing cytosolic calcium by blocking VOCC and inhibiting the intracellular release of calcium through IP_3_ receptor; opening K^+^ channels (K_Ca_, Kir, K_ATP_, Kv); and activating the muscarinic and β2-adrenoreceptor pathways [47]. A *Jasonia glutinosa* extract produces a vasorelaxant effect by preventing increases in cytosolic calcium, mainly by blocking L-type calcium channels [26].

The endothelium is important for vasorelaxation and, of particular note, also for angiogenesis in tissue repair. Our extract produces vasorelaxation by activating the NO/cGMP pathway and by cellular hyperpolarization by opening endothelial IK_Ca_ and SK_Ca_ channels. These signalling pathways not only play a role in endothelium-dependent vascular relaxation but also are important in neo-angiogenesis, as indicated elsewhere [48,49]. For this reason, it would be interesting to study whether *H. stoechas* promotes angiogenesis, particularly during tissue repair. In fact, there are already healing creams containing an extract of this plant. It is tempting to speculate that the healing effect could rely on the antioxidant and vasorelaxing properties of *H. stoechas*, which may help to improve blood circulation in the damaged tissue and may also help to vascularize the tissue by promoting angiogenesis.

## 5. Conclusions

In this study, a methanolic extract of *H. stoechas* has been shown to relax vascular smooth muscle mediated by the endothelium-dependent NO/cGMP pathway and endothelial IK_Ca_ and SK_Ca_ channels. Moreover, the extract’s relaxing actions also involves an endothelium-independent mechanism as it blocks increases in intracellular Ca^2+^ through the inhibition of Ca^2+^ influx via ROCCs or VDCCs as well as the Ca^2+^ release from intracellular stores through the IP_3_ pathway. Considering its relaxing capacity at the endothelial and smooth muscle levels, this medicinal plant could be a useful candidate for the development of herbal medicines for the treatment or prevention of hypertension and other cardiovascular pathologies.

## Data Availability

Data is contained within the article.
